# Development of Straw Mushroom (*Volvariella volvacea*)–Based Broth and Crackers: Nutritional, Microbial, and Antioxidant Evaluation

**DOI:** 10.1155/tswj/9575690

**Published:** 2025-10-23

**Authors:** Feda Anisah Makkiyah, Clarissa Regina Andrestia, Rafi Umar Raihan, Karina, Erna Harfiani, Tri Faranita, Meiskha Bahar

**Affiliations:** ^1^Department of Neurosurgery, Faculty of Medicine, UPN Veteran Jakarta, South Jakarta, Indonesia; ^2^Undergraduate Student of Medical, Faculty of Medicine, UPN Veteran Jakarta, South Jakarta, Indonesia; ^3^Department of Reconstructive Plastic Surgery, Faculty of Medicine, UPN Veteran Jakarta, South Jakarta, Indonesia; ^4^Department of Pharmacology, Faculty of Medicine, UPN Veteran Jakarta, South Jakarta, Indonesia; ^5^Department of Pediatrics, Faculty of Medicine, UPN Veteran Jakarta, South Jakarta, Indonesia; ^6^Department of Microbiology, Faculty of Medicine, UPN Veteran Jakarta, South Jakarta, Indonesia

**Keywords:** antioxidant activity, functional food, monosodium glutamate, mushrooms, savory snacks, *Volvariella volvacea*

## Abstract

Straw mushroom (*Volvariella volvacea*) may offer a natural, flavorful alternative to synthetic food additives such as monosodium glutamate (MSG). This study was aimed at developing safe and nutritious straw mushroom–based broths and crackers that meet consumer demands for healthier snack options without compromising taste. Broth formulations with 25%, 50%, and 75% mushroom content and crackers containing 50, 100, and 150 g mushroom powder were produced using a freeze-drying technique. All samples, including controls without mushrooms, were analyzed for microbial safety (total plate count [TPC] and mold yeast count [MYC]), nutritional composition, and antioxidant capacity (*p* < 0.05). Results showed that all broth and cracker formulations met BPOM (Indonesian Agency of Drug and Food Control) microbial safety standards, with higher mushroom concentrations, with the 75% broth achieving the highest value (2.86 ± 0.02* μ*mol TE/g DW), suggesting enhanced potential health benefits due to higher antioxidant content. Cracker formulations demonstrated nutritional profiles with moisture (2.16%–6.28%), ash (3.76%–4.39%), protein (12.36%–15.39%), fat (12.77%–15.74%), and carbohydrate (61.73%–65.98%). Microbial contamination in all cracker samples remained within safe limits. Among the cracker variants, formulation F2 (100 g mushroom powder) was optimal based on nutritional value and microbial safety. In conclusion, both straw mushroom–based broths and crackers demonstrated promising nutritional quality, safety, and antioxidant potential, supporting their viability as healthier alternatives to MSG-containing snacks.

## 1. Introduction

Rapid urban development in Indonesia has led to significant shifts in dietary habits, particularly in urban areas. Increasingly, people are adopting Western-style diets characterized by high-calorie, high-fat foods rich in artificial flavorings and simple carbohydrates but low in fiber and protein. These dietary trends have contributed to rising rates of noncommunicable diseases such as diabetes, obesity, and cardiovascular disease—conditions linked to poor nutrition and excessive intake of food additives and sugars [1, 2].

Among the most widely used additives is monosodium glutamate (MSG), which enhances savory flavor but has raised health concerns when consumed in excess. While MSG is generally recognized as safe within recommended limits, excessive and prolonged intake may be associated with symptoms such as headaches, increased sodium levels, and other adverse effects [3, 4]. Public health guidelines—including those from the World Health Organization (WHO) and the Indonesian Ministry of Health—have issued recommendations to limit MSG and sugar consumption. However, adherence remains low, and processed foods continue to dominate consumer preferences due to their convenience and taste appeal [5].

In response, there is growing interest in functional food products that offer health benefits beyond basic nutrition. Mushrooms, particularly edible varieties like straw mushrooms (*Volvariella volvacea*), are increasingly recognized for their potential in this area. They are naturally rich in protein, dietary fiber, essential vitamins and minerals, and bioactive compounds such as flavonoids and phenolics, which possess antioxidant properties [6, 7]. Moreover, the presence of glutamic acid in straw mushrooms provides a natural umami flavor, making them a promising alternative to synthetic flavor enhancers like MSG [8].

Straw mushrooms have demonstrated superior nutritional qualities; for example, their protein content is reported to be 19%–35% higher than that of staple foods like rice and wheat [8]. Additionally, the antioxidant compounds found in mushrooms can help neutralize free radicals, reducing oxidative stress and supporting cellular health. Incorporating mushrooms into food formulations not only improves nutritional value but also aligns with clean-label and health-oriented food trends seen globally [9].

Preserving the functional and nutritional quality of mushrooms during processing is essential. Freeze-drying is a preferred technique, as it maintains the integrity of heat-sensitive compounds such as antioxidants and polysaccharides better than conventional drying methods. Studies on oyster and *Inonotus* mushrooms have confirmed that freeze-drying yields higher antioxidant retention and total phenolic content compared to other drying processes [10, 11].

This study is aimed at developing freeze-dried straw mushroom–based broth and crackers; evaluating their nutritional content, antioxidant potential, and microbial safety; and identifying optimal formulations for health-oriented food innovations. These products are intended to provide safe, flavorful, and nutritious alternatives to processed snacks and flavor enhancers, contributing to improved public health outcomes and supporting the development of sustainable, functional foods in Indonesia.

## 2. Material and Methods

### 2.1. Chemical Materials

Chemicals and reagents used in this study were grouped according to their function. For microbial testing, the following materials were employed: Plate Count Agar (PCA) and Potato Dextrose Agar (GranuCult, Merck), 0.9% sodium chloride (NaCl) solution (Braun), 70% ethanol (OneMed), chloramphenicol (Sanbe), and Trolox (Sigma-Aldrich). For proximate and antioxidant analyses, the reagents included mercury(II) oxide (HgO), potassium sulfate (K_2_SO_4_), sulfuric acid (H_2_SO_4_), sodium hydroxide (NaOH), sodium sulfite (Na_2_SO_3_), boric acid (H_3_BO_3_), methyl red, methylene blue, hydrochloric acid (HCl), *n-*hexane, and 0.9% NaCl. All chemicals used were of analytical grade.

### 2.2. Sample Collection and Additives

Fresh *Volvariella volvacea* mushrooms were collected in July 2023 and processed at the Biology Laboratory, Department of Pharmacy, Faculty of Medicine, UPN Veteran Jakarta. Upon receipt, the mushrooms were manually sorted to remove damaged or spoiled specimens, thoroughly cleaned under running potable water to eliminate surface debris, and then sanitized using a 0.9% NaCl solution to reduce microbial load. After draining, the cleaned mushrooms were sliced uniformly and subjected to freeze-drying to preserve their nutritional and antioxidant properties before formulation.

Ingredient used in the preparation of crackers includes wheat flour, baking soda, baking powder, salt, bouillon powder, ground pepper, margarine, water, cornstarch, and skim milk, all of which were procured from certified local markets in South Jakarta. Additional ingredients used in broth formulations–carrots (*Daucus carota*), shallots (*Allium cepa*), garlic (*Allium sativum*), and nutmeg (*Myristica fragrans* Houtt.)—were sourced from Parung District, Bogor, West Java, Indonesia. All plant-based ingredients were washed and peeled where appropriate prior to use.

### 2.3. Freeze-Drying of Broth Material

Straw mushrooms, shallots, garlic, and carrots were cut into small and thin pieces to facilitate the drying process. They were then placed in a freezer at −60°C to −80°C for 24 h to freeze the water. After freezing, the frozen material was put into a vacuum chamber maintained at a level of 2.5 mbar. The temperature inside the vacuum chamber slowly increases until it reaches 38°C to reduce the surrounding pressure. This pressure reduction process causes the water contained in the ingredients to evaporate through the sublimation process, allowing the ingredients to dry completely.

### 2.4. Formulation of Broth Mushroom

The formulations of straw mushroom broth by modifying the study of oyster mushroom broth of Suci [12]. The main ingredient, straw mushroom, used three concentration variations (percentage) shown in Table 1, while the additional ingredients used the same concentration for the three main ingredient formulations.

### 2.5. Manufacturing Process of Crackers

The formulation of straw mushroom crackers refers to Restyawati [13] with modification. Crackers were prepared in three different formulation treatments, as detailed in Table 2. Each treatment was produced in triplicate batches to ensure repeatability and statistical reliability.

The ingredients include baking powder, baking soda, ground pepper, both powder, salt, skimmed milk, corn flour, water, margarine, and wheat flour. The production began by mixing baking soda, salt, baking powder, margarine, ground pepper, skimmed milk, and broth powder using an electric mixer until homogenous. Subsequently, wheat flour, corn flour, straw mushroom powder, and finely chopped celery were added and mixed until a smooth dough formed.

The dough was then rolled out and shaped using a round mold with a diameter of 4 cm. Crackers were placed on a stainless steel perforated baking tray to ensure even heat distribution and baked in a convection oven at 160°C for 15 min.

After baking, the crackers were cooled at room temperature (±25°C) for 30 min on wire racks to prevent moisture buildup. Once cooled, they were packed in airtight polypropylene plastic zip-lock bags and stored at ambient room temperature in a dry, shaded place until further analysis.

### 2.6. Microbial Analysis

Microbial contamination testing was performed using the total plate count (TPC) and mold yeast count (MYC) methods on straw mushroom broth and crackers, in duplicate. A growth medium was prepared by mixing 29 g of media powder with 2650 mL of sterile distilled water, heated while stirring, and sterilized in an autoclave at 121°C for 15 min.

For dilution, each sample was dissolved in a 0.9% NaCl solution to reach a concentration of 10^2^. Then, 1 mL of the solution was placed in a petri dish containing PCA and incubated at 37°C for 24–72 h for TPC and 5 days for MYC.

This study is a true experimental study conducted in vitro, analyzing microorganism growth according to the guidelines from the Indonesian Food and Drug Administration (BPOM). The number of microbial colonies was calculated as per the *Manual of Bacteriological Analysis*.

 $$\begin{array}{c} \text{CFU}/\text{mL}=\frac{\text{number of colonies on all plates}\times \text{dilution factor}}{\text{solution volume}}. \end{array}$$

### 2.7. Proximate Analysis

The Association of Official Analytical Chemist method was used to determine the chemical composition (moisture ash, protein, fat, and carbohydrate) of crackers. Gravimetric methods were used to determine the moisture and ash content. The Kjeldahl method was used to determine the crude protein content. The Soxhlet method was used to determine the crude fat content. Total carbohydrate content was calculated by difference [14].

### 2.8. Antioxidant Activity by DPPH (2,2-Diphenyl-1-Picrylhydrazyl) Assay

The procedure for antioxidant activity was described by Salazar-Aranda et al. [15]. To prepare the samples, we began by dissolving 10 mg of straw mushroom broth powder in 1 mL of DMSO, ensuring thorough dissolution through sonication. We then created a 125 mM DPPH stock solution by dissolving 2.5 mg of DPPH powder in ethanol to achieve a final volume of 50 mL, which was stored in a dark container to maintain its stability.

For the antioxidant testing, we added 100 *μ*L of each prepared sample to a 96-well microplate, followed by 100 *μ*L of the DPPH solution. The microplate was incubated in the dark at room temperature for 30 min to allow for the reaction to occur. After incubation, we measured the absorbance at 517 nm using an ELISA reader. To quantify the antioxidant activity, Trolox was used as a standard at concentrations ranging from 5 to 50 mM, with the results expressed as micromoles of Trolox equivalent per gram of dry weight. This structured approach allows for a clear assessment of the antioxidant potential of the samples.

### 2.9. Statistical Analysis

The best cracker formulation was determined using the exponential comparison method (ECM), a multicriteria decision-making technique commonly applied in decision support systems [16]. The ECM in this study was designed based on nutritional priorities and food safety indicators, specifically proximate composition and microbial contamination results. Criteria were assigned weights through expert judgment by a panel of food science researchers, reflecting the relative importance of each parameter (e.g., higher weight for protein content and microbial safety).

Each parameter was ranked, and a weighted score was calculated by multiplying the rank by its assigned weight. The final score for each formulation was obtained by summing these weighted values. The formulation with the lowest total ECM score was selected as the optimal formulation [17].

Statistical analysis of the mushroom broth data was performed using IBM SPSS software Version 22. The Shapiro–Wilk test was used to assess data normality (as the sample size was ≤ 50). If data were normally distributed (*p* > 0.05), a one-way ANOVA was used to test for significant differences between groups. If the data were not normally distributed (*p* ≤ 0.05), the Kruskal–Wallis test was applied instead. A *p* value less than 0.05 (*α* = 0.05) was considered statistically significant.

## 3. Results

### 3.1. Characteristic of Straw Mushroom Broth and Crackers

**Figure 1:**
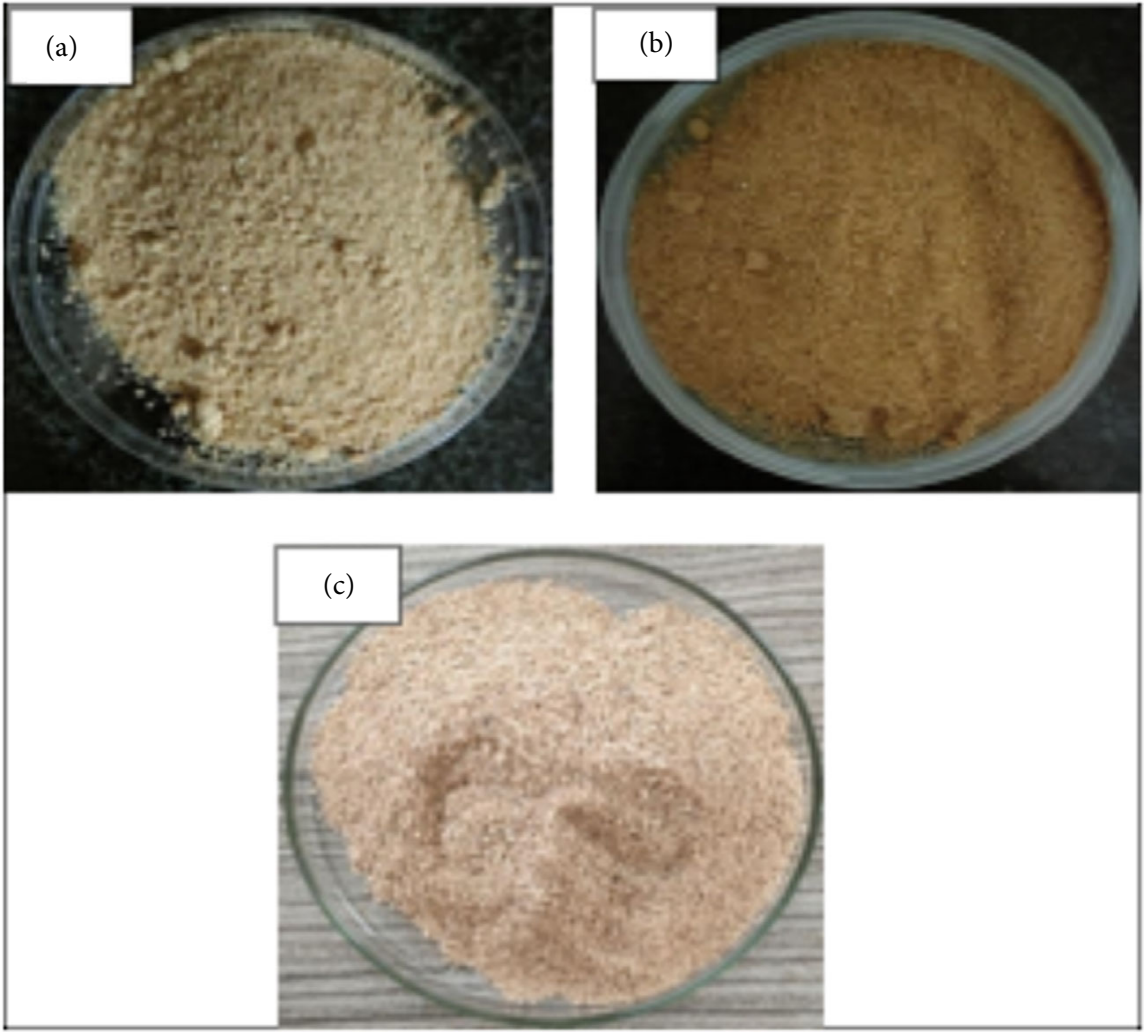
Characteristics of straw mushroom broth in formulation: (a) 25%, (b) 50%, and (c) 75%.

Each formulation of straw mushroom broth showed a different appearance (as shown in Figure 1). The 25%, 50%, and 75% formulations each gave a different color, which were brownish yellow, light brown, and reddish brown, respectively. The addition of additional ingredients to the broth formulation is expected to create a balanced flavor. The addition of garlic and shallots is intended to enhance the flavor and enhance palatability of the broth. The addition of carrot is shown to give a touch of natural sweetness, considering that the broth formulation does not contain sugar as an additional ingredient. Nutmeg is used to give a distinctive aroma.

**Figure 2:**
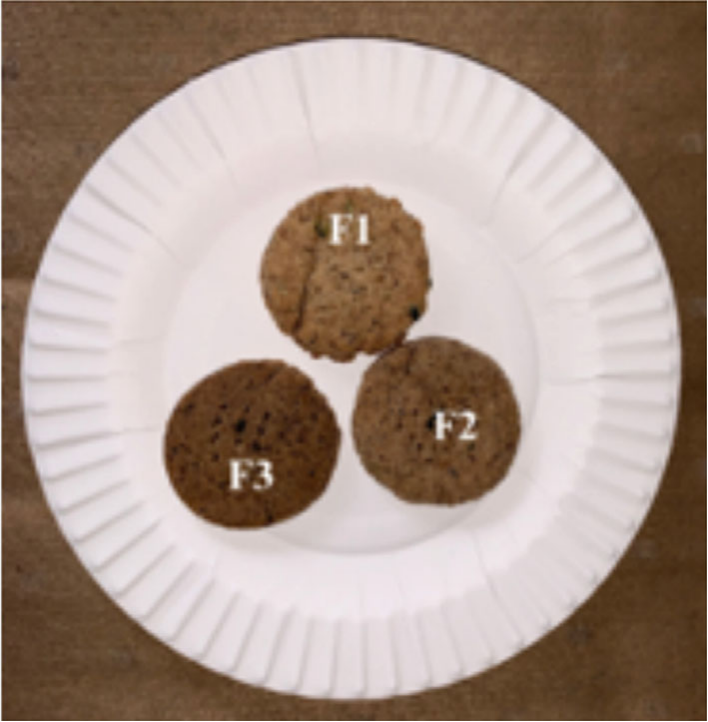
Characteristics of straw mushroom crackers.

The production of crackers (F1, F2, and F3) resulted is a different number of products, between 60 and 75 pieces. Each piece of crackers weighs between 3 and 4 g. The colors of the crackers look darker along with the addition of straw mushroom powder. The appearance of crackers with 50 g (F1), 100 g (F2), and 150 g (F3) of straw mushroom formulation is presented in Figure 2.

### 3.2. Parameter Testing on Broth

#### 3.2.1. Microbial Contamination

Microbial analysis was performed using the TPC and MYC methods, as shown in Table 3. The TPC results indicated that all straw mushroom broth formulations had microbial levels below the maximum permissible limits set by the Indonesian National Agency of Drug and Food Control (BPOM, 2019), which is 1 × 10^6^ CFU/g. Similarly, international standards such as those from the International Commission on Microbiological Specifications for Foods (ICMSF, 2002) also set general microbial limits for ready-to-eat or dried food products within the range of 10^5^–10^6^ CFU/g for TPC, aligning with our findings.

The 25% formulation exhibited the highest TPC at 1.92 × 10^5^ CFU/g, followed by the 75% and 50% formulations at 0.3 × 10^5^ and 0.02 × 10^5^ CFU/g, respectively. All values remained well within acceptable safety thresholds. The MYC results also showed compliance with BPOM standards (1 × 10^3^ CFU/g), with the highest count found in the 75% formulation (0.65 × 10^3^ CFU/g), followed by the 50%, and the lowest in the 25% formulation. Colony growth of straw mushrooms broth formulation is shown in Figure S1 (in supporting information).

#### 3.2.2. Antioxidant Activity by DPPH Assay

The antioxidant capacity based on DPPH radical in straw mushroom broth formulation is shown in Table 4. Antioxidant activity increased with concentration for all formulations. At 5 ppm, no significant difference was observed among the formulations (*p* > 0.05). However, beginning at 10 ppm, the 75% formulation showed significantly higher TEAC values compared with the 25% and 50% formulations (*p* < 0.05). This trend continued at higher concentrations, where the 75% formulation consistently demonstrated superior antioxidant activity. At 25% ppm, the 75% formulation achieved the highest TEAC value (2.86 ± 0.02* μ*mol TE/g DW), which was significantly greater than the 25% and 50% formulations (both 2.64 *μ*mol TE/g DW, *p* < 0.05).

### 3.3. Parameter Testing on Crackers

#### 3.3.1. Proximate Content and Microbial Contamination

The proximate content and TPC of straw mushroom crackers are shown in Table 5, and the colony growth with TPC of straw mushroom crackers is shown in Figure S2. The addition of straw mushroom powder significantly affected protein and moisture contents (*p* < 0.05). F3 had the highest protein content (15.39%), which was significantly higher than F2 (14.42%) and F1 (12.36%). Moisture content was lowest in F1 (2.16%), significantly lower than F2 (3.40%) and F3 (6.28%). Ash content was highest in F2 (4.39%), which differed significantly from F1 (3.76%) and F3 (3.83%). Fat content was significantly lower in F3 (12.77%) compared with F1 and F2, while carbohydrate levels were significantly higher in F1 (65.98%) compared with F2 and F3.

Microbial contamination (TPC) results showed that all cracker formulations complied with the Indonesian National Standard (SNI) requirement of ≤1 × 10^4^ CFU/g. Among the formulations, F1 exhibited the highest microbial load (1 × 10^4^ CFU/g), which was significantly greater that F2 (9.4 × 10^3^ CFU/g) and F3 (9.9 × 10^3^ CFU/g). These findings indicate that all formulations are microbiologically safe, with FS and F3 showing slightly better microbial quality [18].

### 3.4. The Selected Formulation

The selection of the optimal straw mushroom cracker formulation was carried out using the ECM, a decision-support tool commonly used in multicriteria analysis. This method allows for a structured evaluation by combining various performance indicators into a single weighted score.

In this study, six criteria were evaluated (moisture, ash, protein, fat, carbohydrate content, and TPC for microbial contamination). These parameters were selected based on their relevance to product quality, nutritional value, and safety, as defined by the SNI quality standards and supported by relevant food science literature.

Each criterion was assigned a weight (5) reflecting its relative importance in determining product quality. Moisture (25%) and protein (25%) were given the highest weights due to their critical roles in shelf life and nutritional value, and TPC (20%) was emphasized for safety compliance. Then, lower weights were assigned to ash (10%), fat (10%), and carbohydrate (10%), reflecting secondary influence in the final evaluation.

The ranking for each formulation (F1, F2, and F3) under each parameter was determined by performance, with Rank 1 representing the most desirable value (lowest moisture or TPC, highest protein) and Rank 3 representing the least desirable. These ranks were multiplied by the corresponding weights to generate a weighted score for each parameter, which were then summed to yield a total score for each formulation.

As shown in Table 6, F2 achieved the lowest total score (1.90), indicating its superior overall balance across key attributes. It performed well in protein content and microbial safety while maintaining acceptable levels of fat and moisture. F3, although highest in protein, was penalized by its higher moisture content. F1 showed advantages in moisture and carbohydrate levels but ranked lowest in TPC and protein, which are essential indicators for shelf life and nutritional value.

The ECM framework thus enabled a quantitative and transparent comparison of formulations by integrating both nutritional metrics and safety parameters. The selection of F2 as the optimal formulation reflects a balanced trade-off between high nutritional quality, good functional properties, and microbiological safety, aligning with both consumer expectations and regulatory standards.

## 4. Discussions

Straw mushrooms contain a natural compound called glutamic acid, which imparts a savory taste to broth. This makes them a suitable alternative to harmful ingredients like MSG in cooking. Additionally, mushrooms are low in calories yet high in nutritional content, offering various health benefits when used in food. They provide essential nutrients, including carbohydrates (8.7%), protein (26.49%), calcium (0.75%), phosphorus (30%), potassium (44%), vitamins from the B complex, and essential amino acids [19–21]. Straw mushrooms not only provide essential nutrients but also contain secondary metabolites with antibacterial properties. These compounds can inhibit the growth of several types of bacteria, including *Staphylococcus aureus*, *Klebsiella pneumoniae*, *Pseudomonas aeruginosa*, and *Streptococcus pyogenes* [22]. Additionally, straw mushrooms are rich in terpenoid compounds, which have potential as anticancer agents [23].

Freeze-drying is a process that removes water from frozen materials without thawing them, which helps maintain their quality and stability. This method is ideal for sensitive materials and yields high-quality dried mushrooms. However, it typically requires longer drying times and can be more expensive. To enhance drying speed and energy efficiency, combination methods such as microwave drying, ultrasound, or infrared radiation can be employed. Freeze-drying effectively preserves lower moisture content along with high levels of protein, ash, and fiber [24–27].

The microbial analysis of the straw mushroom broth formulations, assessed through TPC and MYC, demonstrated that all samples were within safe consumption limits based on national and international food safety standards. The highest TPC was recorded in the 25% formulation (1.92 × 10^5^ CFU/g), followed by the 75% (0.3 × 10^5^ CFU/g), and the lowest in the 50% formulation (0.02 × 10^5^ CFU/g). These values comply with the maximum allowable microbial limits established by BPOM (2009), which is 1 × 10^6^ CFU/g, as well as international benchmarks such as those provided by the ICMSF (2002), which accept TPC levels up to 10^5^–10^6^ CFU/g for ready-to-eat or dehydrated food products [28].

The MYC values also remained within acceptable limits, with the highest count found in the 75% formulation 0.65 × 10^3^ CFU/g, followed by the 50%, and the lowest in the 25% formulation. All values were below the maximum permitted MYC of 1 × 10^3^ CFU/g, as outlined by BPOM (2019) [28]. These findings indicate that the straw mushroom broth formulations are microbiologically safe and meet both national and global standards for food hygiene.

Differences in TPC values across formulations, as well as in comparison with literature data, can be explained by the influence of ingredient composition and processing methods. Formulations with higher proportions of raw vegetables or mushrooms, such as the 25% and 75% variants, may contribute more natural microbial load due to the presence of native microflora. On the other hand, the 50% formulation appears to have had an optimal balance of solid and liquid components, potentially enhancing the efficiency of microbial reduction during processing. This suggests that ingredient ratio plays a significant role in the final microbial quality by affecting water activity, nutrient availability, and the surface area exposed during drying.

Furthermore, the low microbial counts observed across all samples can be largely attributed to the freeze-drying technique applied during broth preparation. In this process, ingredients were frozen at 60°C–80°C for 24 h, followed by sublimation under a vacuum of 2.5 mbar with a gradual increase in temperature to 38°C. This method reduces water content without passing through the liquid phase, thereby limiting microbial survival and growth. Compared to conventional drying methods, freeze-drying has been shown to achieve superior microbial reduction while preserving the sensory and nutritional properties of food [29, 30]. These characteristics make freeze-drying particularly suitable for the development of shelf-stable, microbiologically safe food products such as instant broths.

Mushrooms are excellent sources of antioxidants, essential for protecting the body against free radicals. The antioxidant activity of straw mushroom broth increased with higher concentrations, indicating its strong potential due to polyphenol content. The freeze-drying method also plays a crucial role in enhancing these properties. This supports findings by Shams et al., which showed that freeze-drying produced higher antioxidant values in *Agaricus bisporus* mushrooms compared to cinerary drying, with DPPH EC50 values of 0.0694 mg/mL and metal-reducing power IC50 values of 30.986 mg/mL [10]. This was also shown by the research of Koffi et al. that freeze-drying on *Volvariella volvacea* has the best antioxidant capacity with DPPH scavenging (EC_50_10.2 ± 0.1 mg/mL) and FRAP (EC_50_10.1 ± 0.1 mg/mL) values [27]. The antioxidant activity of *Volvariella volvacea* showed the highest antioxidant activity compared to the other four types of mushrooms in the research of Tepsongkroh et al., but when compared based on the drying method, tray drying gave higher antioxidant activity than freeze-drying [31]. This is because during thermal drying, peptides with high molecular weight are bound to the product so that brown melanoidins are formed; they may be responsible for high antioxidant activity.

The proximate composition and microbial quality of the straw mushroom crackers reveal important insights into both their nutritional value and food safety. Among the three formulations (F1, F2, and F3), increasing the proportion of straw mushroom powder led to a general rise in moisture, ash, and protein content, while fat and carbohydrate levels showed a declining trend. Notably, all cracker formulations met the microbial safety requirements according to the SNI, with TPC below the permissible limit of 1 × 10^$^ CFU/g, confirming the products' suitability for consumption [18].

From a nutritional perspective, the increase in protein content, particularly in formulation F3 (15.39%), is beneficial, as dietary protein plays a key role in the growth, maintenance, and repair of body tissues. This level compares favorably to commercial cracker products, which typically range from 3% to 14.6% protein. The enrichment from straw mushrooms enhances the amino acid profile and makes these crackers a viable plant-based protein source, particularly for vegetarian or protein-conscious consumers.

Fat content, although slightly decreased with higher mushroom concentration, remained within the acceptable range. While fat contributes essential fatty acids and enhances flavor and texture, excessive fat intake is associated with higher caloric density (9 kcal/g) and an increased risk of obesity and cardiovascular diseases [32]. In this study, lower fat levels, especially in F3, may contribute to improved storage stability by reducing susceptibility to rancidity. This aligns with the goal of developing a healthier, shelf-stable snack alternative.

The carbohydrate content, primarily derived from starch and dietary fiber in wheat flour and straw mushrooms, was the dominant macronutrient in all formulations, ranging from 65.98% in F1 to 61.73% in F3. Despite no added sugar, these values reflect a meaningful contribution of complex carbohydrates, which are digested more slowly and help regulate blood glucose levels. Notably, resistant starches and mushroom-derived fibers may enhance satiety and reduce the risk of overconsumption. Al-Mana and Robertson emphasized the role of starch-based carbohydrates in appetite regulation and energy balance, suggesting that such formulations could support weight management [33].

Ash content, often associated with the total mineral content, increased with higher mushroom powder incorporation. Although a higher ash level may indicate improved mineral intake, excessive levels could also reflect interference with baking performance and raise concerns about potential contaminants. In this study, ash content remained within reasonable levels, supporting the added nutritional value of mushroom inclusion without compromising product quality.

The moisture content also increased with mushroom addition, with F3 slightly exceeding the recommended limit (6.28% vs. the 5% SNI maximum). While this may reduce shelf life by creating conditions favorable to microbial growth, the accompanying TPC results suggest that microbial contamination was still well controlled, likely due to effective drying and processing conditions.

In terms of functional properties, changes in water absorption index (WAI) and water solubility index (WSI), though not detailed in this section, are influenced by the fiber and polysaccharide content of straw mushrooms. Mushroom cell walls contain *β*-glucans, mannans, and chitin, which are hydrophilic polysaccharides that can bind water and swell, increasing the WAI. However, these same components are less soluble, contributing to a decrease in WSI. The balance between WAI and WSI affects the texture, mouthfeel, and rehydration behavior of the crackers. A higher WAI may improve softness and palatability, while a lower WSI can enhance structural integrity and reduce crumbliness during packaging and storage [34, 35].

Overall, the proximate composition and functional attributes of these straw mushroom crackers highlight their potential as nutrient-dense, microbiologically safe, and functionally stable snack products. They offer a promising alternative to conventional crackers, particularly for consumers seeking higher protein, complex carbohydrates, and plant-based functional ingredients.

### 4.1. Research Limitations

Based on the results of the research that has been carried out, there are several limitations that need to be considered. Some of the limitations of the study include the following:

1. In this study, no hedonic quality test was carried out. Therefore, it is not yet known if sensory evaluation or assessment of taste and organoleptical impression on straw mushroom broth formulations is possible.
2. In this study, no bacterial inhibition test was conducted on the straw mushroom broth formulations, so that it does not know the evaluation of the extent to which a food product prevents or inhibit the growth of *Salmonella*, *Enterobacteriaceae*, and *Clostridium perfringens* that may endanger the damage or health of costumers.

## 5. Conclusion

Straw mushroom (*Volvariella volvacea*) has great potential to be used as a healthy food ingredient, such as broth or crackers, because it meets the criteria of processed food standards regulated by BPOM and SNI. The high antioxidant activity of all formulations and microbial contamination analysis based on TPC and MYC obtained the best CFU value in the 50% formulation. Meanwhile, crackers from three formulations met the quality requirements of CFU values based on SNI, and it was concluded that the selected formulation through the ECM was F2 crackers with 3.40% water content, 4.39% ash content, 14.42% protein content, 15.32% fat content, and 62.46% carbohydrate content. This finding has the potential to be developed as a product, especially broth as a safe and nutritious food flavoring, as well as crackers that can be a healthy snack, especially for diet users. Further research is expected to conduct organoleptic tests to determine the quality of color, aroma, taste, and smell of the product so that it can estimate the public acceptance of the product.

## Figures and Tables

**Table 1:** Composition of straw mushroom broth formulations.

**Formulations**	**Main ingredient straw mushroom (%)**	**Additional ingredients**			
		**Garlic (%)**	**Shallot (%)**	**Carrots (%)**	**Nutmeg (%)**
25%	25				
50%	50	30	25	20	25
75%	75				

**Table 2:** Formulation of straw mushroom crackers.

**Materials**	**Formulations**		
	**F1 (g)**	**F2 (g)**	**F3 (g)**
Wheat flour	100	100	100
Straw mushroom powder	50	100	150
Baking soda	1	1	1
Baking powder	1	1	1
Salt	3	3	3
Powdered broth	2	2	2
Ground pepper	2	2	2
Margarine	40	40	40
Water	34	34	34
Cornstarch	20	20	20
Skim milk	10	10	10
Celery	1	1	1

**Table 3:** Microbial contamination results using the TPC test.

**Formulations**	**TPC (CFU/g)**	**MYC (CFU/g)**
25%	1.92 × 10^5^	0.6 × 10^3^
50%	0.02 × 10^5^	0.61 × 10^3^
75%	0.3 × 10^5^	0.65 × 10^3^
BPOM quality	Max. 1 × 10^6^	Max. 1 × 10^4^

**Table 4:** Antioxidant activity of straw mushroom broth.

**Concentrations (ppm)**	**TEAC of formulation mushroom (*μ*mol TE/g DW)**		
	**25%**	**50%**	**75%**
5	2.13 ± 0.00^a^	2.11 ± 0.00^a^	2.20 ± 0.02^a^
10	2.19 ± 0.01^a^	2.19 ± 0.0^a^	2.36 ± 0.01^b^
15	2.27 ± 0.01^a^	2.31 ± 0.02^a^	2.48 ± 0.01^b^
20	2.34 ± 0.08^a^	2.52 ± 0.01^b^	2.61 ± 0.02^c^
25	2.64 ± 0.03^b^	2.64 ± 0.01^b^	2.86 ± 0.02^c^

*Note:* Results are expressed as mean ± SD (*n* = 3) at each concentration. Different superscript letters in the same row indicate significant difference among formulations (*p* < 0.05) based on the one-way ANOVA test.

Abbreviations: *μ*mol TE/g DW, micromoles Trolox equivalent per gram dry weight; ppm, parts per million; TEAC, Trolox equivalent antioxidant capacity.

**Table 5:** Proximate content and TPC of cracker straw mushroom.

**Formulation**	**Component**					
	**Moisture (% ** **w**/**w)**	**Ash (% ** **w**/**w)**	**Protein (% ** **w**/**w)**	**Fat (% ** **w**/**w)**	**Carbohydrate (% ** **w**/**w)**	**TPC (CFU/g)**
F1	2.16^a^	3.76^a^	12.36^a^	15.74^b^	65.98^a^	1 × 10^4^^b^
F2	3.40^b^	4.39^b^	14.42^b^	15.32^b^	62.47^b^	9.4 × 10^3^^a^
F3	6.28^c^	3.83^a^	15.39^c^	12.77^a^	61.73^b^	9.9 × 10^3^^a^
Quality requirement	Max. 5%	—	Max. 4.5%	—	—	1 × 10^4^

*Note:* Values are mean of triplicates. Different superscript letters in the same row indicate significant difference among formulations (*p* < 0.05) based on the one-way ANOVA test.

Abbreviation: TPC, total plate count.

**Table 6:** Multiattribute evaluation of straw mushroom cracker formulations using the ECM.

**Parameter**	**Weight (%)**	**Component alternative scores**					
		**F1**		**F2**		**F3**	
		**Rank**	**Score**	**Rank**	**Score**	**Rank**	**Score**
Nutritional content							
Moisture	25	1	0.25	2	0.50	3	0.75
Ash	10	1	0.10	3	0.30	2	0.20
Protein	25	3	0.75	2	0.50	1	0.25
Fat	10	3	0.30	2	0.20	1	0.10
Carbohydrate	10	1	0.10	2	0.20	3	0.30
Microbial contamination							
Total plate count (TPC)	20	3	0.60	1	0.20	2	0.40
Total score	—	—	2.10	—	1.90	—	2.00
Overall rank	—	—	3	**—**	1	2	—

*Note:* Weighted evaluation of straw mushroom cracker formulation (F1, F2, and F3) using the ECM. Weights were assigned based on regulatory significance and consumer relevance. Each parameter was ranked (1 = *best*, 3 = *worst*), multiplied by its assigned weight, and summed to produce a total score. The formulation with the lowest score (F2) was selected as the most optimal based on balanced nutritional quality and microbial safety.

## Data Availability

Data is available upon request from the authors.

## Supporting Information

Supporting Information Additional supporting information can be found online in the Supporting Information section. The supporting information provide additional experimental data supporting the results of this study. Figure S1 presents the colony growth of straw mushroom broth formulation, which was determined using both the TPC and MYC methods. The TPC results are shown at concentrations of (a) 25%, (b) 50%, and (c) 75%, while the MYC results are presented at (d) 25%, (e) 50%, and (f) 75%. Figure S2 shows the total plate count (TPC) of straw mushroom crackers. These supporting information figures provide further evidence of microbial growth patterns and quality evaluation, complementing the findings discussed in the main text.
