# Supplementary material for: Development of Straw Mushroom (Volvariella volvacea)–Based Broth and Crackers: Nutritional, Microbial, and Antioxidant Evaluation
Source: ScientificWorldJournal. 2025 Oct 23;2025:9575690. doi: 10.1155/tswj/9575690 (PMC12575036; doi:10.1155/tswj/9575690)
Supplement: Supporting Information — Additional supporting information can be found online in the Supporting Information section. The supporting information provide additional experimental data supporting the results of this study. Figure S1 presents the colony growth of straw mushroom broth formulation, which was determined using both the TPC and MYC methods. The TPC results are shown at concentrations of (a) 25%, (b) 50%, and (c) 75%, while the MYC results are presented at (d) 25%, (e) 50%, and (f) 75%. Figure S2 shows the total plate count (TPC) of straw mushroom crackers. These supporting information figures provide further evidence of microbial growth patterns and quality evaluation, complementing the findings discussed in the main text. [file 9575690.f1.docx]

**Bacterial colonies**

a).

b).

c).

d).

e).

f).

**mold colonies**

**Figure S1.** Colony growth of straw mushrooms broth formulation was determined based on the TPC and MYC method. TPC results are a). 25%, b). 50%, and c). 75%, while MYC results are d). 25%, e). 50%, and f). 75%.

**Figure S2.** TPC of straw mushroom crackers.

c).
